# Effects of Nefopam on Postoperative Analgesia in Operating Room-Extubated Patients Undergoing Living Donor Liver Transplantation: A Propensity Score-Matched Analysis

**DOI:** 10.3390/life15040662

**Published:** 2025-04-17

**Authors:** Min Suk Chae, Jin-Oh Jeong, Kyung Kwan Lee, Wonwoo Jeong, Young Wook Moon, Ji Young Min

**Affiliations:** 1 Department of Anesthesiology and Pain Medicine, Seoul St. Mary’s Hospital, College of Medicine, The Catholic University of Korea, Seoul 06591, Republic of Korea; shscms@catholic.ac.kr; 2 Wake Forest Institute for Regenerative Medicine, Wake Forest School of Medicine, Winston-Salem, NC 27157, USA; jijeong@wakehealth.edu (J.-O.J.); kylee@wakehealth.edu (K.K.L.); wjeong@wakehealth.edu (W.J.); 3 US Research and Production Team, CGBIO USA, Winston-Salem, NC 27101, USA; moon@cgbio.co.k; 4 Department of Anesthesiology and Pain Medicine, Eunpyeong St. Mary’s Hospital, College of Medicine, The Catholic University of Korea, 1021 Tongil-ro, Eunpyeong-gu, Seoul 03312, Republic of Korea

**Keywords:** enhanced recovery after surgery, liver transplantation, nefopam, pain, postoperative analgesia

## Abstract

In patients undergoing living donor liver transplantation (LDLT) with immediate postoperative extubation in the operating room (OR), rapid recovery of consciousness and spontaneous ventilation are essential, requiring effective analgesia without compromising respiratory function. This study evaluated whether intraoperative nefopam administration improves early postoperative pain control and reduces opioid consumption in this physiologically distinct population. A retrospective cohort of 376 adult LDLT recipients who met the criteria for OR extubation was analyzed. After propensity score matching, 182 patients who received intraoperative nefopam were compared with 182 matched controls. Pain intensity was measured using the visual analog scale (VAS), and total fentanyl consumption and opioid-related complications were recorded over the first 24 h postoperatively. Nefopam administration was associated with significantly lower VAS scores during the first 12 h after surgery (*p* < 0.001) and reduced 24 h fentanyl consumption (53.2 ± 20.8 mL vs. 58.6 ± 27.5 mL, *p* = 0.035). No serious adverse effects related to nefopam were observed. The incidence of postoperative nausea and vomiting did not differ significantly between the groups. These findings indicate that nefopam offers effective early analgesia and an opioid-sparing effect in LDLT recipients undergoing OR extubation, suggesting its clinical utility as a component of multimodal analgesia in this high-risk group. Although the reduction in opioid use did not translate into a decreased incidence of opioid-related complications, the favorable safety profile and analgesic efficacy of nefopam support further investigation through prospective trials to define its role in enhanced recovery protocols for OR-extubated LDLT recipients.

## 1. Introduction

Living donor liver transplantation (LDLT) has become an essential therapeutic option for patients with end-stage liver disease, offering improved access and survival benefits compared to deceased donor liver transplantation (DDLT) [1]. In regions with limited availability of deceased donor organs, LDLT plays a pivotal role in expanding transplant opportunities. However, the surgical complexity of LDLT—characterized by extensive hepatobiliary dissection, large abdominal incisions, and prolonged operative time—frequently results in significant postoperative pain [2]. Inadequately managed pain not only impairs patient comfort but also adversely affects respiratory mechanics, delays mobilization, and contributes to hemodynamic instability, all of which may negatively impact graft function and postoperative recovery [3].

Enhanced recovery after surgery (ERAS) protocols are increasingly being adopted in liver transplantation to improve postoperative outcomes and accelerate recovery [4]. A core component of ERAS is early extubation, ideally performed in the operating room (OR extubation), which has been associated with reduced ventilator-associated complications, shorter ICU stays, and faster functional recovery [5,6]. Importantly, the ability to achieve rapid and effective postoperative pain control without inducing respiratory depression or excessive sedation is one of the key determinants of successful OR extubation in LDLT recipients [7]. These patients must resume spontaneous breathing and initiate early mobilization shortly after extubation, making the optimization of analgesia both a clinical and logistical challenge [8].

Although opioids are widely used as first-line agents for postoperative pain management, their dose-dependent adverse effects—such as respiratory depression, sedation, ileus, and immunosuppression—can be particularly detrimental in LDLT patients, especially those extubated in the OR [9]. Furthermore, many conventional non-opioid analgesics are either contraindicated or used with caution in this population. Nonsteroidal anti-inflammatory drugs (NSAIDs) pose a risk of renal dysfunction and bleeding, while acetaminophen may worsen hepatic injury or interact with immunosuppressants [3]. In addition, regional anesthesia techniques (e.g., epidural or paravertebral blocks), often utilized in abdominal surgery, are frequently avoided in LDLT due to concerns about coagulopathy and potential hemodynamic compromise [10]. These limitations underscore the need for safe and effective opioid-sparing alternatives tailored to the physiologic needs of LDLT recipients undergoing fast-track recovery.

Nefopam is a centrally acting non-opioid analgesic that has gained attention as a potential component of multimodal analgesia. It exerts its analgesic effect by inhibiting the reuptake of serotonin, norepinephrine, and dopamine, thereby modulating pain perception via central mechanisms [11]. Importantly, nefopam does not impair platelet function or renal perfusion and lacks the sedative and respiratory depressive properties typical of opioids. These characteristics make nefopam a particularly attractive option for LDLT patients, who require a rapid return of consciousness and respiratory function post-extubation. Although nefopam has demonstrated opioid-sparing effects and a favorable safety profile in various general surgical populations, its efficacy and tolerability in OR-extubated liver transplant recipients—especially in the context of altered hepatic metabolism—have not been clearly established [12,13].

This study aimed to investigate the clinical utility of nefopam in LDLT recipients who undergo early extubation in the operating room. We evaluated whether intraoperative nefopam administration improves postoperative pain control and reduces opioid consumption during the critical early recovery period. Additionally, we examined its impact on opioid-related complications, such as postoperative nausea and vomiting (PONV), to determine its potential role as part of a multimodal analgesic strategy tailored to the unique needs of this high-risk population.

## 2. Patients and Methods

### 2.1. Ethical Considerations

This investigation was designed as a retrospective observational cohort study and conducted according to the ethical standards established by the Declaration of Helsinki. Ethical approval was granted by the Institutional Review Board and Ethics Committee at Seoul St. Mary’s Hospital (approval ID: KC21IRSI0576; approval date: 17 September 2021). Due to the retrospective nature of the study, obtaining informed consent from individual patients was exempted. All results presented in this manuscript adhere to the recommendations specified by the Strengthening the Reporting of Observational Studies in Epidemiology (STROBE) guidelines (File S1).

### 2.2. Study Population

The study included adult patients aged ≥ 19 years who underwent elective primary LDLT with planned immediate OR extubation between January 2015 and December 2022. Patients eligible for inclusion were those successfully extubated in the operating room postoperatively, had complete medical records documenting pain scores and opioid consumption, and met predefined clinical and anesthetic criteria. Detailed inclusion criteria are summarized in Supplementary Table S1. Exclusion criteria encompassed patients requiring reintubation within 24 h post-surgery, those with a history of chronic pain or preoperative opioid use, known contraindications to nefopam (e.g., hypersensitivity or a history of seizures) [10], or incomplete data on key outcomes. Additionally, patients with significant postoperative complications, such as massive bleeding or the need for reoperation, as well as those with a history of cardiovascular diseases—due to nefopam’s potential cardiovascular effects—were excluded [10]. To ensure homogeneity, patients who underwent deceased donor liver transplantation or ABO-mismatched liver transplantation were also excluded. To ensure homogeneity, patients who underwent deceased donor liver transplantation or ABO-mismatched liver transplantation were also excluded.

**Figure 1:**
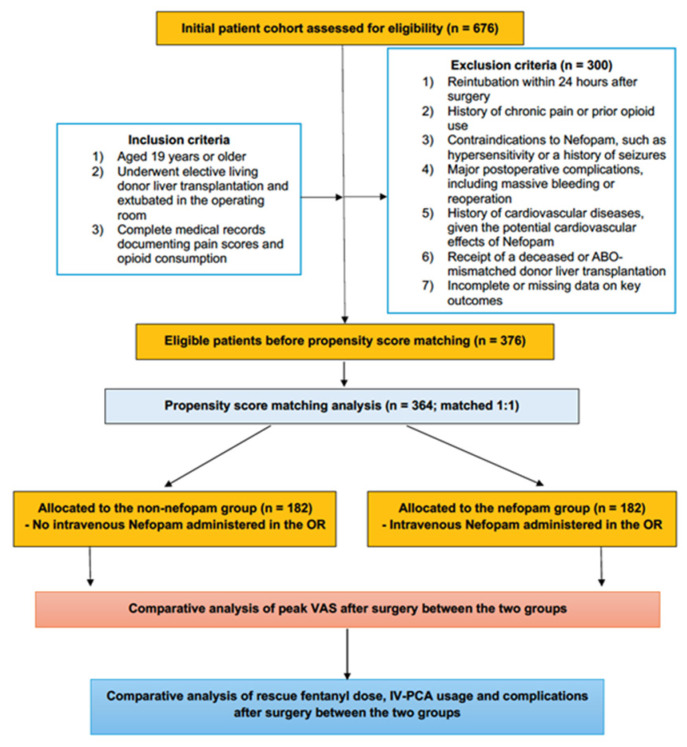
Consort diagram.

From an initial cohort of 676 patients, 376 met the eligibility criteria after applying the predefined exclusion parameters. Propensity score (PS) matching was employed to balance baseline characteristics between the groups, resulting in a final cohort of 364 patients, evenly divided between the nefopam group (n = 182) and the non-nefopam group (n = 182) for the final analysis (Figure 1).

### 2.3. Surgery and Anesthesia

The surgical technique consisted of a J-shaped subcostal incision, and the right hepatic lobe was harvested using the piggyback method. Reconstruction of the middle hepatic vein was conducted to optimize venous drainage. Anastomoses of vessels and bile ducts were subsequently performed, and graft perfusion and vascular patency were confirmed by intraoperative Doppler ultrasonography. General anesthesia was maintained with desflurane, remifentanil, and rocuronium, accompanied by continuous invasive hemodynamic monitoring, including arterial blood pressure, central venous pressure, and cardiac output. Any intraoperative hemodynamic instability was addressed with personalized fluid management and targeted vasopressor therapy. Diuretics were also administered as necessary to manage fluid overload or decreased urinary output, thereby maintaining stable intraoperative conditions throughout surgery [14,15].

Eligibility for operating room extubation was determined by attending anesthesiologists based on predefined criteria [16], while ventilatory function required a tidal volume > 5 mL/kg, a respiratory rate < 25 breaths/min, and ETCO_2_ levels between 30–40 mmHg. Stable hemodynamics with minimal vasopressor support (norepinephrine < 0.1 μg/kg/min) and complete neuromuscular recovery were essential prerequisites. Patients also needed to exhibit intact protective reflexes, stable metabolic parameters (pH > 7.25, normal electrolyte levels), and a core temperature ≥ 35.5 °C. Extubation was performed only when there were no significant bleeding issues or vascular concerns. Following extubation, the patients were transferred to the intensive care unit (ICU) for postoperative monitoring and care.

### 2.4. Postoperative Opioid-Based Pain Management

Postoperative pain control was initiated during surgical site closure approximately 30 min prior to planned extubation in the operating room, with a 100 μg fentanyl infusion. Upon transfer to the ICU, a fentanyl solution (1500 μg diluted in 200 mL of normal saline) was administered via infusion, with doses adjusted as needed to maintain pain relief (visual analog scale (VAS) ≤ 4), as determined by the attending ICU physician.

Pain levels were monitored using the VAS at 1, 4, 8, 12, and 24 h postoperatively. All fentanyl dosing decisions were made exclusively by the attending ICU physician, who was not involved in the study, ensuring unbiased pain management. The nursing staff closely observed patients for potential opioid-related side effects, including nausea, vomiting, respiratory depression, excessive sedation, constipation, and pruritus, enabling prompt interventions to ensure patient safety and comfort.

### 2.5. Nefopam as an Adjuvant Non-Opioid Analgesia

For patients scheduled for operating room extubation, nefopam (Myungmoon Pharm. Co., Seoul, Republic of Korea) was utilized as a non-opioid adjunct to manage postoperative pain. Its selection was based on its established safety profile in hepatectomy patients, offering a safer alternative to acetaminophen and NSAIDs [17,18,19]. A 20 mg dose of nefopam, diluted in 100 mL of normal saline, was administered intravenously over 15 min just prior to the patient’s emergence from anesthesia.

To ensure safety, the pharmacologic properties of nefopam were carefully considered, including its onset of action (15–30 min), peak effect (30–60 min), and duration of action (4–6 h) [20]. The patients were closely monitored for 24 h postoperatively for adverse effects such as nausea, vomiting, tachycardia, dizziness, excessive sweating, and localized reactions at the infusion site, ensuring timely intervention if needed.

### 2.6. Outcome Measures

The primary outcome of the study was postoperative pain assessment, measured using the visual analog scale (VAS) at 1, 4, 8, 12, and 24 h after surgery. Secondary outcomes included total fentanyl consumption during the first 24 h postoperatively and the incidence of opioid-related side effects, specifically nausea and vomiting. Pain intensity was expressed as mean VAS scores, while fentanyl consumption was quantified as the total volume administered.

### 2.7. Clinical Variables for Propensity Score Matching Analysis

Propensity score (PS) matching was conducted to reduce potential confounding and ensure balanced baseline characteristics between the nefopam and non-nefopam groups. Propensity scores were calculated using logistic regression analysis, incorporating a comprehensive set of recipient demographic, clinical, laboratory, intraoperative, and donor-related variables. Recipient demographic and clinical variables included age, sex, body mass index (BMI), pre-existing comorbidities (hypertension and diabetes mellitus), Model for End-Stage Liver Disease (MELD) score, presence of significant clinical complications (varices and ascites ≥ 1 L), and echocardiographic parameters, including left ventricular ejection fraction and presence of diastolic dysfunction. Preoperative laboratory parameters assessed were white blood cell count, neutrophil and lymphocyte percentages, hematocrit, platelet count, serum albumin, ammonia, total bilirubin, liver enzyme levels (aspartate aminotransferase (AST) and alanine aminotransferase (ALT)), electrolytes (sodium, potassium, calcium), and coagulation status, as reflected by the international normalized ratio (INR). Intraoperative variables included operation duration, hourly rates of fluid infusion and urine output, intraoperative transfusion requirements (packed red blood cells and fresh frozen plasma), and average intraoperative vital signs (systolic blood pressure, diastolic blood pressure, and heart rate). The donor graft-related variables analyzed were donor age, sex, BMI, graft weight, graft-to-recipient weight ratio (GRWR), graft fatty percentage, and total ischemic time.

### 2.8. Statistical Analysis

The Shapiro–Wilk test was employed to evaluate the normality of the continuous variables. Continuous data are presented as medians with interquartile ranges, while categorical data are reported as counts and percentages. PS were calculated using logistic regression analysis incorporating recipient demographic, clinical, laboratory, intraoperative, and donor-related covariates. One-to-one nearest-neighbor matching was performed using a greedy algorithm without replacement to minimize confounding factors and ensure group balance. Perioperative recipient and donor graft variables were analyzed using Student’s *t*-test or the Mann–Whitney U test for continuous data, depending on their distribution. For categorical data, either the χ^2^ test or Fisher’s exact test was used based on data distribution and sample size. Statistical significance was set at a *p*-value of <0.05. All analyses were conducted using SPSS for Windows (version 24.0; IBM Corp., Armonk, NY, USA) and Microsoft Excel.

## 3. Results

### 3.1. Demographic Variables

This study included 376 adult patients who underwent elective LDLT. The majority of the patients were male (n = 273, 72.6%), with a mean age of 53.8 ± 9.6 years and an average body mass index (BMI) of 24.8 ± 4.0 kg/m^2^. Hepatitis B was the most common underlying etiology (n = 236, 62.8%), followed by alcohol-related liver disease (n = 95, 25.3%), hepatitis C (n = 27, 7.2%), drug-induced liver disease (n = 10, 2.7%), and autoimmune liver disease (n = 8, 2.1%). The cohort had a mean MELD score of 16.7 ± 12.0 points, reflecting the severity of liver dysfunction in the study population.

### 3.2. Clinical Characteristics in the Nefopam and Non-Nefopam Groups Before and After PS Matching

Table 1 summarizes the demographic and clinical characteristics of recipients and donors in the nefopam and non-nefopam groups before and after PS matching. Prior to matching, some baseline imbalances were observed between the groups, including a higher proportion of males and a lower average BMI in the nefopam group, as well as differences in MELD scores and certain laboratory parameters, such as total bilirubin levels and hematocrit.

After PS matching, 182 patients were included in each group, achieving balanced distributions across all key variables. No significant differences were observed in demographic characteristics, comorbidities, MELD scores, laboratory findings, or intraoperative variables such as operation time and transfusion requirements. The donor graft variables, including graft-recipient weight ratio and total ischemic time, were also comparable. These results confirm that PS matching successfully minimized baseline differences, ensuring robust comparative analysis between the two groups.

### 3.3. Postoperative Pain, Opioid Use, and Complications in PS-Matched Patients

**Figure 2:**
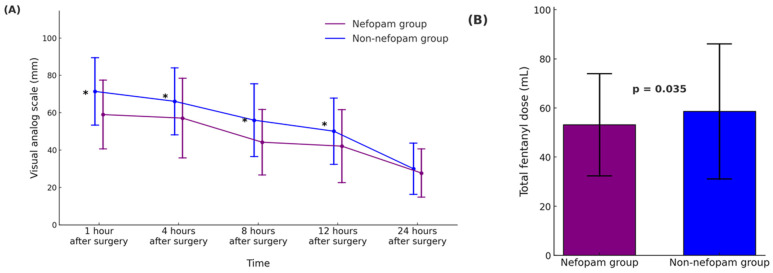
Postoperative pain intensity and opioid consumption during the first 24 h after surgery in operating room (OR)-extubated living donor liver transplantation (LDLT) recipients. (**A**) The time course of the postoperative pain scores, assessed using the visual analog scale (VAS, 0–100 mm), is presented for the nefopam and non-nefopam groups. Pain scores are shown as mean ± standard deviation (SD), and asterisks (*) denote statistically significant differences between the groups (*p* < 0.001). (**B**) Total intravenous fentanyl dose (mL) administered over the first 24 h postoperatively, presented as mean ± SD. Error bars represent standard deviation. The nefopam group demonstrated significantly lower fentanyl consumption compared to the non-nefopam group (*p* = 0.035). In both panels, the nefopam group is represented in purple, and the non-nefopam group in blue. Abbreviations: LDLT, living donor liver transplantation; VAS, visual analog scale.

Table 2 and Figure 2 summarize the outcomes related to postoperative pain, opioid consumption, and complications in the propensity score-matched nefopam and non-nefopam groups. In Figure 2A, pain intensity measured using the visual analog scale (VAS) was significantly lower in the nefopam group at 1, 4, 8, and 12 h after surgery (*p* < 0.001 for all time points). However, no significant difference was observed 24 h postoperatively (*p* = 0.103). Figure 2B demonstrates that total fentanyl consumption during the first 24 h was significantly lower in the nefopam group (53.2 ± 20.8 mL) compared to the non-nefopam group (58.6 ± 27.5 mL, *p* = 0.035), indicating reduced opioid requirements in patients receiving nefopam. The incidence of opioid-related side effects, such as nausea and vomiting, was comparable between the groups. Nausea occurred in 4.4% of patients in the nefopam group and 6.0% in the non-nefopam group (*p* = 0.480), while vomiting was observed in 2.2% and 1.6% of patients, respectively (*p* = 0.703).

### 3.4. Nefopam-Related to Complications

Patients in the nefopam group were closely monitored during the first postoperative day for potential adverse effects, including tachycardia, dizziness, sweating, and injection site reactions. No significant complications, such as excessive tachycardia, palpitations, hemodynamic instability, headache, blurred vision, or injection site reactions, were reported. Mild symptoms, such as dizziness or sweating, were observed in 2 patients and resolved spontaneously without requiring any interventions. These findings indicate that nefopam is well tolerated with minimal and self-limiting side effects.

## 4. Discussion

This study demonstrated that nefopam significantly reduced early postoperative pain intensity and opioid consumption in patients undergoing LDLT with OR extubation. Pain scores were notably lower in the nefopam group during the first 12 postoperative hours, and total 24 h fentanyl consumption was significantly reduced, highlighting nefopam’s opioid-sparing potential. Importantly, nefopam was well tolerated, with only minor, transient symptoms such as dizziness and sweating, and no serious cardiovascular or neurologic side effects observed. However, no significant difference in the incidence of PONV was noted between the groups, suggesting that opioid reduction alone may be insufficient to influence PONV in this population. These results indicate the potential relevance of nefopam in the context of the unique clinical characteristics of OR-extubated LDLT recipients.

The clinical relevance of these findings is particularly notable in this physiologically distinct subgroup of liver transplant patients. Unlike DDLT recipients, who often present with higher MELD scores and reduced pain perception due to advanced disease, LDLT recipients undergo transplantation at an earlier stage, with better-preserved hepatic and neurologic function [16,20,21]. As a result, they are more likely to experience heightened postoperative pain and require more intensive analgesic support, particularly during the immediate postoperative period [14,21]. These challenges are particularly pronounced in patients undergoing OR extubation, who must resume spontaneous breathing and initiate early mobilization without the benefit of gradual sedation weaning. As previously discussed, this highlights the need for early, well-balanced analgesia that preserves respiratory function while supporting recovery—key goals of ERAS protocols [22,23].

In this context, nefopam emerges as a practical analgesic option, providing effective pain control with reduced opioid reliance, consistent with prior findings in abdominal and hepatic surgery [19,24,25,26]. LDLT procedures are associated with significant postoperative pain due to extensive dissection and broad surgical fields. Achieving effective analgesia without compromising respiratory or hemodynamic stability remains a critical challenge. Although the opioid-sparing effect of nefopam in this study was modest, even small reductions in opioid use may decrease the risk of dose-dependent complications and support recovery in fast-track perioperative care [27]. The absolute reduction in fentanyl consumption, while statistically significant, may appear numerically limited. However, in LDLT recipients undergoing immediate postoperative extubation—who are particularly vulnerable to opioid-related adverse effects such as respiratory depression, sedation, and delayed mobilization—even incremental reductions may yield clinically meaningful benefits [9,22]. Nevertheless, relatively high postoperative pain scores (VAS > 40 mm) were observed, underscoring the persistent challenge of achieving adequate analgesia in this population. These elevated pain levels likely reflect extensive surgical trauma, hepatobiliary manipulation, and limitations in regional techniques due to coagulopathy. Although intraoperative nefopam contributed to statistically and clinically significant analgesia [28], additional optimization may require adjunctive agents such as intravenous acetaminophen, low-dose ketamine, or lidocaine infusions, as well as novel regional techniques tailored to individual coagulation status. Future studies should investigate comprehensive multimodal strategies to improve pain outcomes in this high-risk surgical group.

While the retrospective design of this study limits definitive causal inference, the observed findings are supported by both the pharmacologic rationale and existing clinical evidence. Nefopam exerts its analgesic effects by centrally inhibiting the reuptake of serotonin, norepinephrine, and dopamine. Its onset of action occurs within 15–30 min of administration, with a half-life of approximately 3–5 h, allowing sustained analgesia during the early postoperative period [11,28,29]. Multiple randomized controlled trials and observational studies of abdominal and hepatic surgery have demonstrated significant early postoperative analgesic effects and reductions in opioid use with intraoperative nefopam administration [19,25,30]. Taken together, these findings provide a pharmacologically plausible and clinically supported basis for nefopam’s integration into perioperative pain management. Prospective randomized controlled trials are necessary to further validate its efficacy and safety in LDLT recipients.

Despite multimodal analgesia increasingly being advocated to mitigate the well-known risks of opioid use [31,32,33], many non-opioid agents also have limitations in this population. NSAIDs and acetaminophen carry risks of nephrotoxicity, hepatotoxicity, and coagulopathy, while regional techniques are often contraindicated due to coagulopathy or hemodynamic instability [34,35,36]. In contrast, nefopam provides effective systemic analgesia without impairing hepatic, renal, or coagulation function. Although it is metabolized via CYP2D6—which may lead to pharmacokinetic variability in patients with altered hepatic function—no such adverse effects were observed in our cohort [19]. However, given nefopam’s pharmacologic profile, caution is still warranted in patients with cardiovascular instability or autonomic dysfunction during the immediate postoperative period.

This study’s findings support nefopam’s role in OR-extubated LDLT recipients, where rapid recovery of consciousness and stable spontaneous ventilation are essential goals [37]. Prior studies have shown that 20 mg of nefopam provides analgesia comparable to 12 mg of morphine [38], reinforcing its inclusion in ERAS-based multimodal pain management strategies. Although PONV incidence was not significantly different between the groups, this may reflect its multifactorial nature. This suggests that opioid reduction alone may be insufficient to address PONV in this patient group, underscoring the need for additional antiemetic strategies or targeted adjunctive measures as part of comprehensive postoperative care. Moreover, as regional anesthesia is often contraindicated in LDLT patients [10], nefopam remains a practical systemic analgesic—particularly when combined with non-sedating agents such as IV acetaminophen or lidocaine. Collectively, these findings support nefopam’s role as a safe, effective, and contextually appropriate adjunct in pain management protocols for OR-extubated LDLT recipients, provided that individualized risk assessment and vigilant perioperative monitoring are maintained.

This study has several limitations. First, as a retrospective observational study utilizing propensity score matching, residual confounding and selection bias remain possible, despite statistical adjustments [39]. Therefore, the causal relationships between nefopam administration and clinical outcomes must be cautiously interpreted. Future prospective randomized controlled trials (RCTs) are essential to confirm these preliminary results rigorously. Second, the single-center study design limits the generalizability of findings, as institutional differences in surgical techniques, anesthetic protocols, and postoperative management may influence outcomes [40]. Multicenter studies are necessary to validate the efficacy and safety of nefopam across various transplant centers. Third, we did not comprehensively assess all potential adverse effects of nefopam, including detailed cardiovascular (e.g., hypertension, tachycardia) or neurologic events [41,42,43], nor did we measure patient-reported outcomes such as analgesic satisfaction or functional recovery scores. Future studies incorporating standardized safety assessments and patient-centered measures are warranted. Finally, our follow-up period was limited to the first 24 postoperative hours, precluding an evaluation of nefopam’s long-term effects on recovery parameters such as mobilization, ICU length of stay, graft function, and systemic inflammation. Prospective studies with longer follow-up durations are required to thoroughly evaluate these clinically relevant outcomes.

## 5. Conclusions

In conclusion, this study’s findings indicate that nefopam significantly decreases early postoperative pain scores and reduces total opioid consumption within the first 24 h after surgery in LDLT recipients undergoing OR extubation, without serious adverse effects. These results support its role as a safe and effective component of multimodal analgesia in this physiologically distinct patient group, where early recovery, stable spontaneous ventilation, and hemodynamic stability are critical. Although its opioid-sparing effect did not significantly reduce the incidence of PONV, nefopam remains a practical systemic analgesic—particularly in settings where regional techniques are contraindicated. Incorporating nefopam into ERAS-based pain management protocols may facilitate faster and safer recovery, provided that individualized risk assessment and vigilant perioperative monitoring are maintained. Future prospective studies should aim to optimize nefopam dosing strategies, determine the ideal timing of administration, and evaluate its long-term impact on recovery trajectories and graft outcomes.

## Figures and Tables

**Table 1:** Baseline characteristics of recipients and donor/graft variables in operating room (OR) extubated LDLT recipients before and after propensity score matching between the nefopam and non-nefopam groups.

	Before Propensity Score Matching				After Propensity Score Matching			
	Nefopam (n = 188)	Non-Nefopam (n = 188)	*p*-Value	SD	Nefopam (n = 182)	Non-Nefopam (n = 182)	*p*-Value	SD
***Preoperative recipient variables***								
Sex; n (%)	141 (75.0%)	132 (70.2%)	0.298	−0.110	135 (74.2%)	130 (71.4%)	0.556	−0.063
Age; years	54.0 (49.0–60.0)	54.0 (48.3–61.0)	0.972	−0.024	54.0 (49.0–60.0)	54.0 (48.8–61.0)	0.990	−0.027
BMI; kg/m^2^	23.9 (22.0–26.5)	24.7 (22.2–27.1)	0.057	−0.195	23.9 (22.0–26.5)	24.6 (22.2–26.9)	0.158	−0.125
Comorbidity								
Hypertension	43 (22.9%)	40 (21.3%)	0.709	0.038	42 (23.1%)	39 (21.4%)	0.705	0.039
Diabetes mellitus	56 (29.8%)	49 (26.1%)	0.421	0.081	54 (29.7%)	47 (25.8%)	0.413	0.084
MELD score; points	12.0 (6.1–22.9)	16.5 (7.6–26.0)	0.094	−0.142	11.9 (6.1–22.5)	15.7 (7.5–25.3)	0.094	−0.148
Complications								
Varix	52 (27.7%)	50 (26.6%)	0.817	0.024	49 (26.9%)	50 (27.5%)	0.906	−0.012
Ascite (≥1 L)	85 (45.2%)	100 (53.2%)	0.122	−0.160	82 (45.1%)	94 (51.6%)	0.208	−0.132
Echocardiography								
Ejection fraction; %	64.4 (62.0–66.8)	64.0 (61.8–66.0)	0.128	0.197	64.4 (62.0–66.7)	64.0 (61.9–66.0)	0.141	0.163
Diastolic dysfunction (≥grade II); n (%)	30 (16.0%)	25 (13.3%)	0.466	0.072	28 (15.4%)	25 (13.7%)	0.656	0.045
Laboratory values								
WBC count; ×10^9^/L	4.5 (3.0–7.4)	5.0 (3.1–8.0)	0.259	−0.157	4.4 (3.0–7.0)	4.9 (3.0–7.7)	0.255	−0.155
Neutrophil; %	63.0 (52.5–76.9)	63.0 (53.3–73.7)	0.892	0.030	62.4 (52.0–76.2)	63.0 (53.2–73.6)	0.861	−0.001
Lymphocyte; %	22.3 (11.3–32.3)	19.9 (11.9–30.9)	0.831	0.012	22.8 (11.8–32.8)	20.2 (11.9–31.0)	0.625	0.036
Hematocrit; %	30.3 (25.3–36.6)	28.6 (24.4–34.3)	0.046	0.196	30.3 (25.4–36.5)	28.7 (24.5–34.6)	0.078	0.177
AST; U/L	50.5 (36.3–78.5)	50.0 (36.0–81.8)	0.987	0.072	50.0 (35.8–72.5)	50.0 (35.0–82.5)	0.897	−0.002
ALT; U/L	33.0 (20.0–60.0)	31.0 (21.0–58.0)	0.815	0.059	33.0 (20.0–59.3)	31.0 (21.0–59.3)	0.969	−0.019
Total bilirubin; mg/dL	2.3 (0.8–12.4)	3.4 (1.1–18.7)	0.034	−0.204	2.3 (0.8–12.3)	3.2 (1.0–18.0)	0.051	−0.182
Sodium; mmol/L	140.0 (136.0–142.0)	139.0 (135.0–142.0)	0.433	0.089	140.0 (136.0–142.0)	139.0 (135.0–142.0)	0.408	0.088
Calcium; mg/dL	8.4 (7.9–8.9)	8.4 (7.9–8.9)	0.366	0.008	8.4 (7.9–8.9)	8.4 (7.9–8.9)	0.453	−0.003
Potassium; mmol/L	3.9 (3.6–4.3)	4.0 (3.6–4.4)	0.494	−0.094	4.0 (3.6–4.3)	4.0 (3.6–4.4)	0.730	−0.009
Albumin; g/dL	3.1 (2.7–3.7)	3.0 (2.6–3.5)	0.180	0.154	3.1 (2.7–3.7)	3.1 (2.6–3.5)	0.344	0.114
Ammonia; μg/dL	94.5 (68.3–146.8)	92.0 (66.0–138.8)	0.371	0.127	95.0 (68.8–144.3)	95.0 (66.0–139.5)	0.469	0.120
Platelet count; ×10^9^/L	67.5 (48.3–103.8)	65.5 (44.0–104.5)	0.537	0.041	66.0 (48.0–103.0)	65.0 (43.8–103.5)	0.586	0.036
INR	1.4 (1.2–2.1)	1.6 (1.3–2.2)	0.083	−0.161	1.4 (1.2–2.0)	1.5 (1.2–2.2)	0.159	−0.156
***Intraoperative recipient variables***								
Operation time; min	475.0 (415.0–535.0)	472.5 (429.3–530.0)	0.695	0.018	475.0 (415.0–535.0)	472.5 (428.0–530.0)	0.587	0.003
Hourly fluid infusion; mL/kg/h	12.5 (9.4–16.2)	12.6 (9.4–17.3)	0.863	0.010	12.5 (9.4–16.2)	12.6 (9.5–17.4)	0.506	−0.015
Hourly urine output; mL/kg/hr	1.3 (0.5–2.2)	1.1 (0.5–1.9)	0.229	0.122	1.3 (0.5–2.2)	1.1 (0.5–1.9)	0.243	0.106
Average of vital signs								
Systolic blood pressure; mmHg	107.3 (98.1–115.8)	104.4 (96.8–113.5)	0.096	0.159	107.1 (98.4–115.6)	104.1 (96.8–113.8)	0.099	0.155
Diastolic blood pressure; mmHg	56.1 (51.0–61.7)	55.8 (49.5–60.9)	0.285	0.127	56.1 (51.0–61.6)	55.8 (49.5–60.8)	0.316	0.113
Heart rate; beats/min	89.3 (81.4–98.3)	89.1 (78.8–100.3)	0.826	0.010	89.3 (81.5–98.3)	89.1 (78.8–100.5)	0.913	−0.013
Transfusion								
Packed red blood cells; units	8.0 (4.0–11.0)	8.0 (4.0–12.0)	0.547	−0.094	7.0 (4.0–11.0)	8.0 (4.0–12.0)	0.483	−0.106
Fresh frozen plasma; units	6.0 (4.0–10.0)	7.0 (4.0–10.0)	0.495	−0.043	6.0 (4.0–10.0)	7.0 (4.0–10.0)	0.391	−0.053
***Donor graft variables***								
Sex; n (%)	139 (73.9%)	132 (70.2%)	0.421	−0.085	133 (73.1%)	130 (71.4%)	0.725	−0.037
Age; years	35.4 (28.0–41.0)	35.4 (26.0–43.0)	0.734	0.046	35.4 (28.0–41.0)	35.4 (26.8–43.0)	0.773	0.044
BMI; kg/m^2^	20.2 (18.9–22.1)	20.2 (19.0–22.2)	0.679	−0.090	20.2 (18.9–22.2)	20.2 (19.0–22.1)	0.740	−0.071
Graft-recipient-weight ratio; %	1.2 (1.1–1.6)	1.3 (1.0–1.6)	0.864	−0.011	1.2 (1.1–1.6)	1.3 (1.1–1.6)	0.849	−0.046
Graft weight; g	866.0 (700.0–1049.5)	862.0 (738.5–1115.0)	0.722	−0.076	863.0 (699.5–1043.0)	866.0 (739.5–1105.0)	0.596	−0.094
Fatty percentage; %	4.9 (0.0–5.0)	4.9 (1.0–5.0)	0.137	−0.059	4.9 (0.0–5.0)	4.9 (1.0–5.0)	0.176	−0.055
Total ischemic time; min	89.0 (63.5–132.0)	83.0 (62.3–138.0)	0.470	0.054	86.5 (63.0–133.5)	83.0 (62.8–139.3)	0.718	0.030

Values are expressed as median (interquartile) and number (percentage). Abbreviations: LDLT, living donor liver transplantation; SD, standard deviation; BMI, body mass index; MELD, Model for End-Stage Liver Disease; AST, aspartate aminotransferase; ALT, alanine aminotransferase; INR, international normalized ratio.

**Table 2:** Comparison of postoperative pain scores, opioid consumption, and complications between the nefopam and non-nefopam groups in propensity score-matched operating room (OR) extubated LDLT recipients.

	Nefopam (n = 182)	Non-Nefopam (n = 182)	*p*-Value
**Visual analog scale; mm**			
1 h after surgery	59.0 ± 18.4	71.4 ± 18.1	<0.001 **
4 h after surgery	57.1 ± 21.3	66.1 ± 17.9	<0.001 **
8 h after surgery	44.2 ± 17.5	56.0 ± 19.5	<0.001 **
12 h after surgery	42.1 ± 19.5	50.1 ± 17.7	<0.001 **
24 h after surgery	27.7 ± 12.9	30.0 ± 13.7	0.103
**Total fentanyl dose for 24 h after surgery; mL**	53.2 ± 20.8	58.6 ± 27.5	0.035 *
**Opioid-related complications**			
Nausea	8 (4.4%)	11 (6.0%)	0.480
Vomiting	4 (2.2%)	3 (1.6%)	0.703

Values are expressed as mean (±standard deviation) and number (percentage). Abbreviations: LDLT, living donor liver transplantation. * *p* < 0.05, ** *p* < 0.001.

## Data Availability

Data are contained within the article. The datasets used and analyzed in the current study are available from the corresponding author upon reasonable request.

## Supplementary Materials

The following supporting information can be downloaded at: https://www.mdpi.com/article/10.3390/life15040662/s1, File S1: STROBE Statement; Table S1: Detailed Inclusion Criteria for the Study.
