# Supplementary material for: Effects of Nefopam on Postoperative Analgesia in Operating Room-Extubated Patients Undergoing Living Donor Liver Transplantation: A Propensity Score-Matched Analysis
Source: Life (Basel). 2025 Apr 17;15(4):662. doi: 10.3390/life15040662 (PMC12029016; doi:10.3390/life15040662)
Supplement: Supplementary file 1 [file life-15-00662-s001.zip › life-3576338-Table S1.pdf]

Table S1. Detailed Inclusion Criteria for the Study

**Category**	**Detailed Criteria**
Age	≥19 years
Surgery type	Elective living donor liver transplantation
Extubation timing	Immediate postoperative extubation in the operating room (OR extubation)
Medical records	Complete postoperative records, including documented pain scores (VAS) and opioid consumption data for first 24 hours
Hemodynamic stability	Stable intraoperative and immediate postoperative hemodynamics, defined as minimal vasopressor use (norepinephrine <0.1 μg/kg/min)
Respiratory parameters	Adequate spontaneous ventilation parameters at extubation:<br>- Tidal volume >5 mL/kg<br>- Respiratory rate <25 breaths/min<br>- $ETCO_2$ 30–40 mmHg
Neuromuscular function	Fully reversed neuromuscular blockade confirmed by Train-of-Four (TOF) monitoring (TOF ratio ≥0.9)
Mental status	Fully awake and responsive (able to follow verbal commands clearly)
Core body temperature	Core temperature ≥35.5°C at the time of extubation
Clinical complications	No significant intraoperative or immediate postoperative complications, including:<br>- Massive hemorrhage<br>- Severe hemodynamic instability<br>- Uncontrolled arrhythmias<br>- Severe metabolic/electrolyte disturbances
